# The Mechanical Properties and In Vitro Biocompatibility of PM-Fabricated Ti-28Nb-35.4Zr Alloy for Orthopedic Implant Applications

**DOI:** 10.3390/ma11040531

**Published:** 2018-03-30

**Authors:** Wei Xu, Ming Li, Cuie Wen, Shaomin Lv, Chengcheng Liu, Xin Lu, Xuanhui Qu

**Affiliations:** 1 Institute for Advanced Materials and Technology, University of Science and Technology Beijing, Beijing 100083, China; xuweicool@126.com (W.X.); g20159107@xs.ustb.edu.cn (M.L.); lsmleon@163.com (S.L.); liucc1988@163.com (C.L.); 2 School of Engineering, RMIT University, 3083 Melbourne, Australia; cuie.wen@rmit.edu.au; 3 Beijing Key Laboratory for Advanced Powder Metallurgy and Particulate Materials, University of Science and Technology Beijing, Beijing 100083, China; 4 Beijing Laboratory of Metallic Materials and Processing for Modern Transportation, University of Science and Technology Beijing, Beijing 100083, China

**Keywords:** Ti-28Nb-35.4 alloy, powder metallurgy, ball milling, mechanical properties, biocompatibility

## Abstract

A biocompatible Ti-28Nb-35.4Zr alloy used as bone implant was fabricated through the powder metallurgy process. The effects of mechanical milling and sintering temperatures on the microstructure and mechanical properties were investigated systematically, before in vitro biocompatibility of full dense Ti-28Nb-35.4Zr alloy was evaluated by cytotoxicity tests. The results show that the mechanical milling and sintering temperatures have significantly effects on the density and mechanical properties of the alloys. The relative density of the alloy fabricated by the atomized powders at 1500 °C is only 83 ± 1.8%, while the relative density of the alloy fabricated by the ball-milled powders can rapidly reach at 96.4 ± 1.3% at 1500 °C. When the temperature was increased to 1550 °C, the alloy fabricated by ball-milled powders achieve full density (relative density is 98.1 ± 1.2%). The PM-fabricated Ti-28Nb-35.4Zr alloy by ball-milled powders at 1550 °C can achieve a wide range of mechanical properties, with a compressive yield strength of 1058 ± 35.1 MPa, elastic modulus of 50.8 ± 3.9 GPa, and hardness of 65.8 ± 1.5 HRA. The in vitro cytotoxicity test suggests that the PM-fabricated Ti-28Nb-35.4Zr alloy by ball-milled powders at 1550 °C has no adverse effects on MC3T3-E1 cells with cytotoxicity ranking of 0 grade, which is nearly close to ELI Ti-6Al-4V or CP Ti. These properties and the net-shape manufacturability makes PM-fabricated Ti-28Nb-35.4Zr alloy a low-cost, highly-biocompatible, Ti-based biomedical alloy.

## 1. Introduction

Metallic biomaterials, which are the materials of choice for orthopedic implants, are fundamental for improve the quality of life and longevity of human beings [1,2]. Many different metallic materials have been used in a variety of applications in the medical field. Specifically, they are used for internal support and biological tissue replacements such as joint replacement, dental roots, orthopedic fixation and stents [3]. The common metals and alloys that are being utilized for biomedical applications include: stainless steels, Co-based alloys, and Ti-based alloys. Ti-based alloys have dominated in the human hard tissue repair and dentistry fields due to high strength, low elastic modulus, and excellent in vivo corrosion and biocompatibility [4]. However, despite their attractive performance, there are still challenges facing their application in artificial joints [5]. These concerns are mainly related to bio-toxicity, the need for lower elastic modulus and better mechanical strength. For instance, Al elements in extra-low interstitial (ELI) Ti-6Al-4V (hereafter all compositions are given in wt %), Ti-5Al-2.5Fe and Ti-6Al-7Nb [6,7,8], which is the most widely applied Ti alloy, are considered to be related to some health-related problems, including Alzheimer disease and neuropathy [9,10]. In addition, the elastic modulus (~110 GPa) of these alloys is still considerably higher than those of the cortical bones (~30 GPa), which results in severe ‘stress shielding’ for implantation failures [11,12]. Hence, considerable amount of effort has been exeerted to develop Al- and V-free lower-modulus β-Ti alloys. 

Notable examples include the United States Food and Drug Administration (FDA)-approved proprietary alloys of Ti-13Nb-13Zr and Ti-12Mo-6Zr-2Fe and the non-proprietary alloy of Ti-15Mo. In particular, Ti-Nb-Zr alloys have received significant attention due to their excellent mechanical compatibility and biocompatibility [13,14,15,16]. The addition of Nb to Ti can stabilize the β phase and results in improved mechanical properties, which also results in improved wear resistance and corrosion resistance [17,18]. Furthermore, the addition of Zr helps in obtaining the solid solution required for achieving the hardness [19,20]. In addition, as reported by references [21,22], the elastic modulus in Ti-Nb and Ti-Zr alloys decreases with an increase in the content of the Nb and Zr within certain limits. Based on these results, Wen et al. [23] designed the new Ti-Nb-Zr alloys using the d-electron design method combined with the molybdenum equivalence (Mo_eq_) and electron-to-atom ratio (e/a) approaches. The results show that the alloys have an excellent combination of mechanical properties and biocompatibility. However, at present these alloys are manufactured by the conventional route (i.e., ingot metallurgy in addition to wrought processing and machining with up to 90% being scrapped), which leads to a high manufacturing cost. Hence, in order to reduce costs and enhance the utilization rate of materials, the powder metallurgy (PM) technique is introduced. PM is an advanced net-shape technique which particularly suits to large volume production and can reduce processing steps, hence reducing cost [24,25,26,27]. It has already been used to synthesize Ti-based alloys by many researchers. For example, Sharma et al. [28] used a powder metallurgy route consisting of mechanical alloys (MA) of the TiH_2_-Nb powder mixture and spark plasma sintering (SPS) to produce Ti-40 mass% Nb alloys. Jia et al. [29] successfully obtained the Ti-22Al-25Nb alloy by PM. Mendes et al. [30] produced the alloy Ti-27Nb-13Zr with low Young’s modulus by PM using powders produced by the hydride-dehydride (HDH) process.

In this study, the Ti-28Nb-35.4Zr alloy is fabricated by PM in order to obtain Ti-based alloy with excellent mechanical compatibility and biocompatibility in addition to further reducing the manufacturing cost. The effects of sintering temperatures and mechanical milling on density, microstructure and mechanical properties are investigated systematically, before the in vitro biocompatibility of the full dense Ti-28Nb-35.4Zr is evaluated preliminarily. We aimed to establish a necessary understanding of the low-cost PM-fabricated Ti-28Nb-35.4Zr alloy for orthopedic implant applications.

## 2. Experimental and Methods

### 2.1. Materials and Sample Preparation

Atomized Ti-28Nb-35.4Zr powders (purity ≥ 99.9%, 75 ≤ particle size ≤ 150 μm) was supplied by the Wen group (RMIT University, Melbourne, Australia), who fabricated the powders using continuous inert gas atomization without the crucible method. The atomized Ti-28Nb-35.4Zr powders were subjected to ball milling for 30 min using a three-dimensional vibration ball milling machine (HSVM, Nanjing Chishun Science and Technology Co., Ltd., Nanjing, China). The frequency of the vibration ball milling machine was 1400 r/min, while the ball-to-powder weight ratio was 3:1. The materials of the balls and jars were stainless steel and GCr15 bearing steel, respectively. The milling process was carried out in a high-purity argon atmosphere, with 2 wt % stearate used as the process control agent (PCA). Atomized powder (AP) and ball-milled powder (BMP) were cold-pressed into cylindrical compacts under 450 MPa. Then isothermal sintering was carried out in the argon (Ar) protection environment and implemented in two steps. Specimens were initially heated to 1000 °C for 2 h at 5 °C/min, before being heated at 2 °C/min to six different temperatures between 1200 °C and 1550 °C for 2 h. This was followed by furnace cooling to room temperature to obtain samples.

### 2.2. Materials Characterization 

The density was measured by the Archimedes method and the relative density was calculated by the following formula:

Relative density = the density/the theoretical density(1)

where the theoretical density is 6.36 g/cm^3^. The hardness was carried out using the HDI-1875 Rockwell hardness tester. Five points were measured, before the average value was calculated. X-ray diffraction (XRD) was performed using a Dmax-RB X-ray diffractometer (Cu Kα, λ = 0.15406 nm, Rigaku, Tokyo, Japan). A JSM-6510V (JEOL, Tokyo, Japan) scanning electron microscope (SEM) was used to analyze the powder morphology of the received atomized powders and ball-milled powders and sintered microstructure. Compression specimens with a gauge size (φ) of 3 mm × 5 mm were fabricated by electric discharge machining and the specimen surface was polished with SiC papers. The compression test was performed on an Instron machine (Instron, Boston, MA, USA) at the strain rate of 2 × 10^−3^ s^−1^ at room temperature. The compressive yield strength and elastic modulus were calculated from the engineering stress strain curves.

### 2.3. In Vitro Biocompatibility Testing

Cytotoxicity tests were carried out with murine osteoblast cells (MC3T3-E1) to examine the in vitro biocompatibility of the Ti-28Nb-35.4Zr alloy. For comparison, the cast ELI Ti-6Al-4V and CP-Ti were studied simultaneously. The cells were cultured in Dulbecco’s modified Eagle’s medium (DMEM, Shanghai solarbio Bioscience and Technology Co., Ltd., Shanghai, China) containing 10% fetal bovine serum (FBS), 100 U/mL penicillin, and 100 μg/mL streptomycin at 37 °C under a humidified atmosphere of air containing 5% CO_2_. The metal samples were cut into discs of 10 mm in diameter and 1 mm in thickness via electric discharge machining, before the surface was polished with SiC papers (grit 400 down to 5000). After this, the samples were cleaned ultrasonically and sterilized for further use. 

In the cytotoxicity test, the extracts were obtained based on the international standard ISO 10993-5 [31]. The cells were incubated by an extraction medium in 96-well plates at the density of 5000 cells per 100 μL. At the desired times (day 1, day 2 and day 3), cells were observed by the optical microscope (LEXT OLS4000, Olympus, Tokyo, Japan), while the 10 μL MTT solution (Shanghai solarbio Bioscience and Technology Co., Ltd., Shanghai, China) was added to each well and were incubated for 4 h. After this, 100 μL of dimethyl sulfoxide (DMSO, Shanghai solarbio Bioscience and Technology Co., Ltd., Shanghai, China) was added to each well, before being incubated for a further 5 min. The absorbance was recorded by a multimode detector on a Synergy HT (BioTek, Winooski, VT, USA) at a wavelength of 570 nm. The cell viability ratio (CVR) was calculated by the formula as follows:

(2) $$CVR=\left(OD_{570nm\;}\mathrm{in}\;\mathrm{experimental}\;\mathrm{extract}/OD_{570nm}\;\mathrm{in}\;\mathrm{control}\;\mathrm{extract}\right)\;\times \;100\%$$

Based on the international standard ISO 10993-5 [31], the cytotoxic level was divided into six groups: 0: ≥100%; I: 75–99%; II: 50–74%; III: 25–49%; IV: 1–24%; V: ≤1%. Cell viability ratio were analyzed using one-way analysis of variance (ANOVA, P < 0.05) followed by the Tukey honestly significant difference (HSD) post-hoc test. P < 0.05 was considered to be statistically significant.

## 3. Results and Discussion

### 3.1. Raw Powder Characterization 

**Figure 1:**
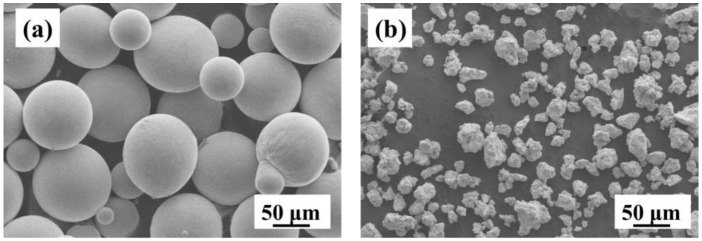
SEM images (800×) of the powders: (**a**) Atomized powders; and (**b**) Ball-milled powders.

Figure 1 depicts the representative SEM micrographs of atomized and ball-milled powders. It can be seen that the atomized powders (Figure 1a) have a typical spherical shape. The particle size of the powders varies from 30.2 to 160.5 μm with an average particles size of approximately 80.5 μm. The morphology of the Ti-Nb-Zr particles after 30 min of ball milling is shown in Figure 1b. The particle size of the ball-milled powders varies from 5.1 to 30.5 μm with an average particle size of approximately 15.2 μm. The shape of the powders becomes irregular, which makes further powder treatment easier (green compaction) as irregular powder particles have higher compressibility and green strength [32]. 

**Figure 2:**
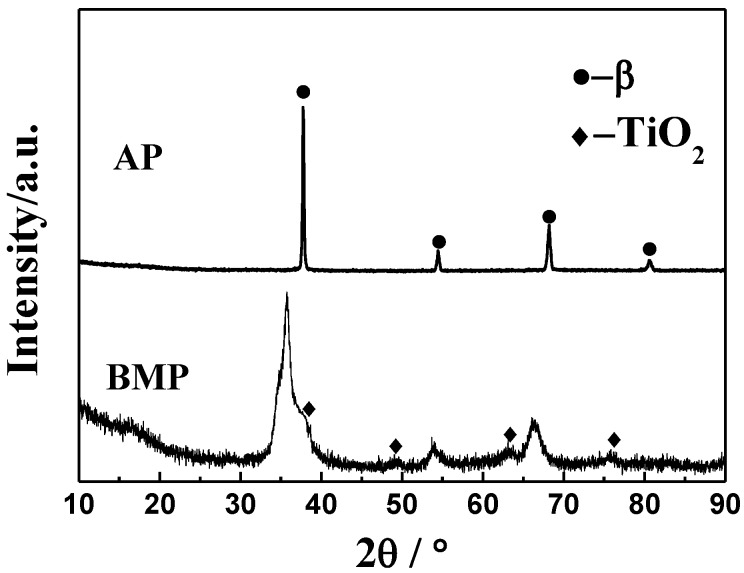
XRD patterns of atomized and ball-milled powders.

Figure 2 shows the XRD patterns of atomized and ball-milled powders. It can be seen that atomized powders consist of a single β-Ti, while the ball-milled powders consist of β-Ti and TiO_2_. TiO_2_ mainly emerges because there is some oxygen introduced during the process of ball milling. In addition, compared with the atomized powders, the diffraction spectrums of the ball-milled powders shows an obvious broadening and moves to low angle, which is associated with the reduction in grain size, increase in lattice distortion and instrumental effects [33]. 

### 3.2. As-Sintered Density

**Figure 3:**
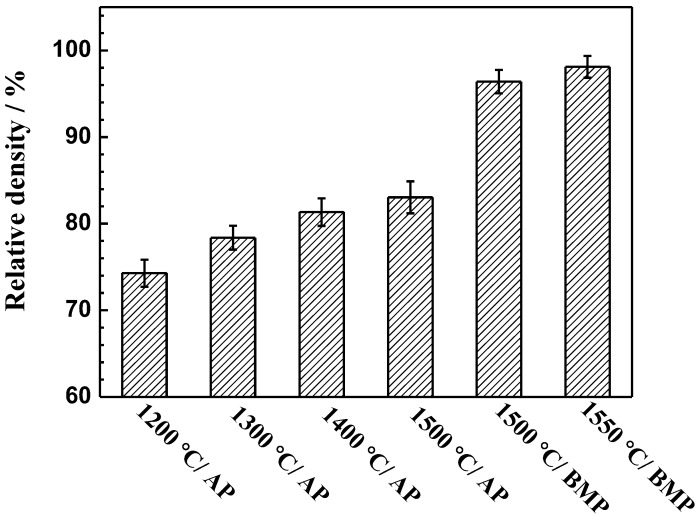
Relative density of Ti-28Nb-35.4Zr alloy prepared with different sintering temperatures and powders.

Figure 3 shows the variation in relative density of samples with respect to sintering temperatures and powders. The relative density strongly depends on both sintering temperatures and powders. For the atomized powder, with an increase in the sintering temperature the relative density of the alloys increases gradually, but it is difficult to achieve a high relative density. The relative density of the alloy sintered at 1500 °C is only 83.1 ± 1.8%. Compared to the samples fabricated by atomized powder, the relative density of the samples fabricated by milled powder at 1500 °C rapidly increases to 96.4 ± 1.3%. When the sintering temperature increases to 1550 °C, the relative density of the alloy reaches 98.1 ± 1.2%.

**Figure 4:**
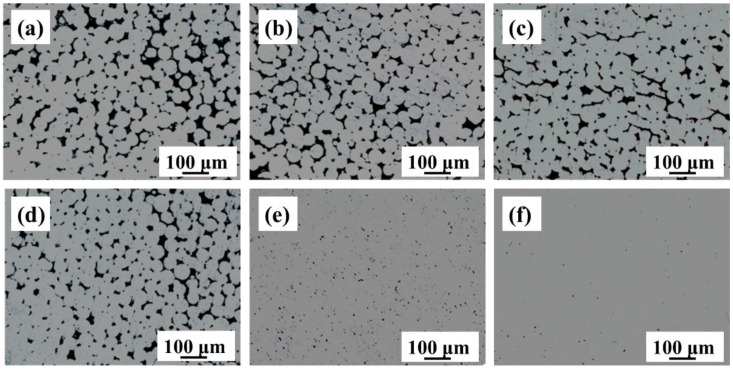
Optical images of Ti-28Nb-35.4Zr prepared with different sintering temperatures and raw powders: (**a**) 1200 °C/AP; (**b**) 1300 °C/AP; (**c**) 1400 °C/AP; (**d**) 1500 °C/AP; (**e**) 1500 °C/BMP and (**f**) 1550 °C/BMP.

Figure 4 shows the optional images of Ti-28Nb-35.4Zr prepared with different sintering temperatures and powders. As shown in Figure 4a–d, the porosity of the samples fabricated by atomized powders decreases gradually with an increase in the sintering temperature. However, there are many pores on the surface of the samples even when they are sintered at 1500 °C. As shown by Figure 4e,f, the porosity of alloy prepared by milled powder reduces significantly, while the alloy becomes close to full density when sintered at 1550 °C. This is mainly associated with the sintering process of powder particles. After milling, the average particle sizes of the powders become smaller and hence, the powders have a high interface energy, which makes the sintering neck form more easily. Therefore, compared with the samples fabricated by atomized powder, the alloys prepared by milled powders have a higher density and therefore higher relative density.

### 3.3. As-Sintered Microstructure

**Figure 5:**
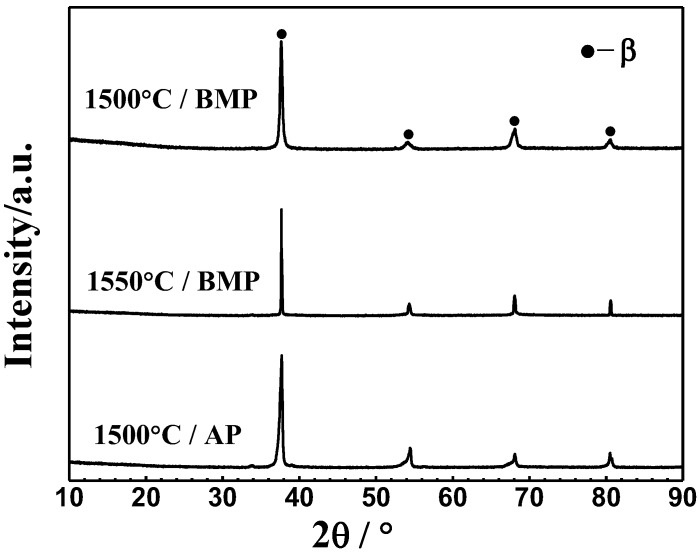
XRD patterns of Ti-28Nb-35.4Zr alloy prepared with different temperatures and powders.

Figure 5 shows the XRD patterns of Ti-28Nb-35.4Zr alloy fabricated by different powders at different sintering temperature. It can be seen that there is no significant difference of XRD patterns of Ti-28Nb-35.4Zr alloy fabricated by different powders and sintering temperatures. The clear diffraction peaks suggest that the samples all have similar phase compositions and mainly consist of single β phases.

**Figure 6:**
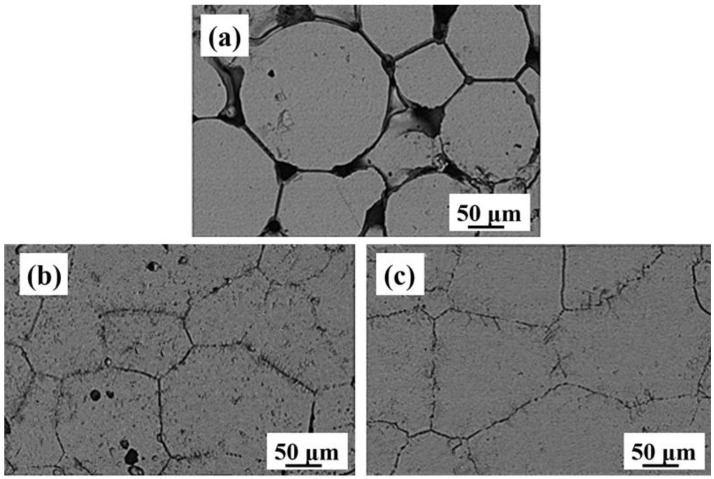
SEM images (800×) of Ti-28Nb-35.4Zr alloy prepared with different sintering temperatures and powders: (**a**) 1500 °C/AP; (**b**) 1500 °C/BMP and (**c**) 1550 °C/BMP.

Figure 6 displays the SEM micrographs of Ti-28Nb-35.4Zr alloy samples sintered at different temperatures with different powders, which was taken under 800× magnification. It can be seen that some pores exist in the alloy fabricated by the atomized powder at 1500 °C and the original powder particle boundaries can be observed clearly. However, the alloy prepared by milled powder at 1500 °C has a few pores, while the alloy becomes nearly fully dense with no obvious pores when the sintering temperature increases to 1550 °C. In addition, as shown in Figure 6b,c, the samples fabricated by the milled powder show a similar microstructure, which is consistent with the XRD results. Both samples consist of a single β phase and the average grain size is about 100 ± 10 μm.

### 3.4. Room-Temperature Mechanical Properties

**Figure 7:**
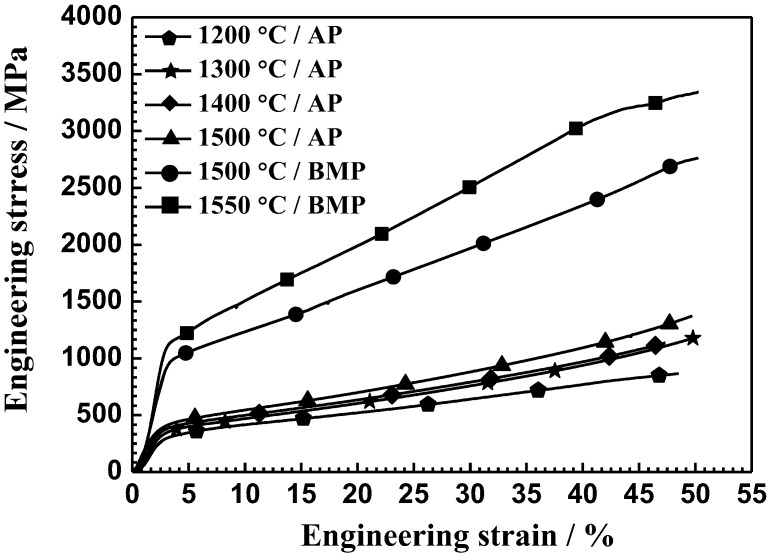
Compressive stress–strain curves of Ti-28Nb-35.4Zr alloy prepared with different sintering temperatures and powders.

Figure 7 shows compressive engineering stress-strain curves of the Ti-28Nb-35.4Zr alloy fabricated by different powders at different sintering temperatures. As shown in Figure 7, the alloys exhibit similar stress–strain behavior and no fracture was observed during their compression process, demonstrating that the alloys exhibit considerable elastic–plastic deformation ability. Compression tests were stopped after reaching a strain value of about 50%.

**Figure 8:**
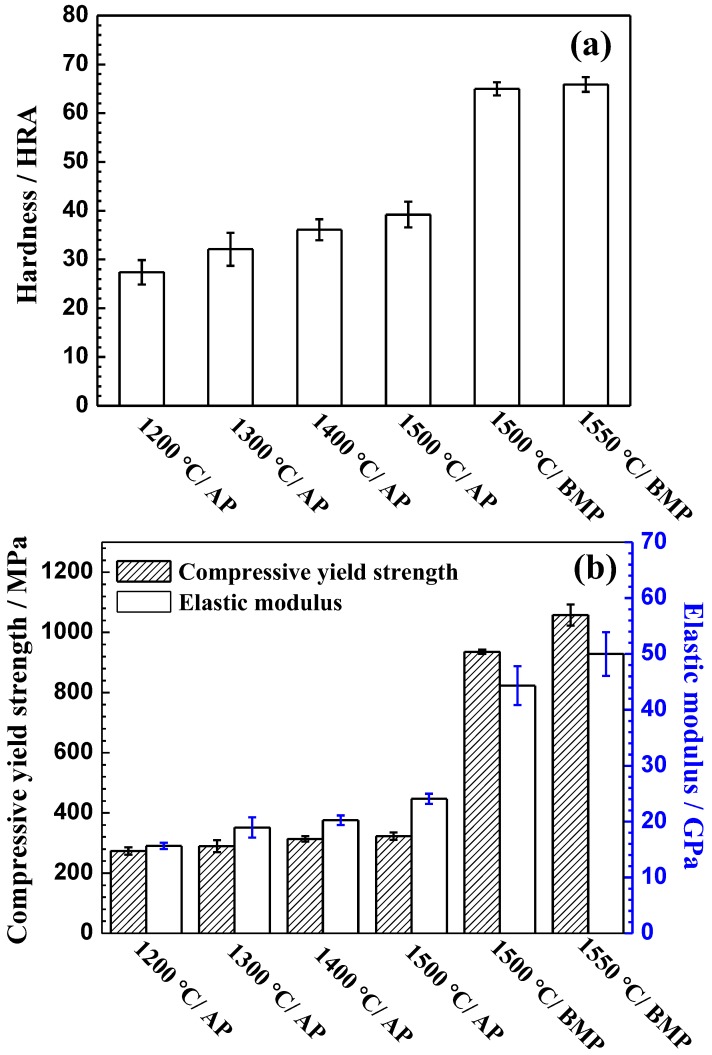
Mechanical properties of Ti-28Nb-34.5Zr alloy prepared with different sintering temperatures and powders: (**a**) Hardness; (**b**) Compressive elastic modulus and yield strength.

The compressive yield strength, elastic modulus and hardness of Ti-28Nb-35.4Zr alloy fabricated by different powders at different sintering temperatures are shown in Figure 8. The hardness, compressive yield strength and elastic modulus of the Ti-28Nb-35.4Zr alloy fabricated atomized powder generally increase with an increase in the sintering temperature, which were in the range of 27.3–39.2 HRA, 273–332 MPa and 15.6–24.0 GPa, respectively. Compared with the alloy fabricated by the atomized powder, the mechanical properties of the alloy prepared by milled powder are significantly improved. The hardness of the alloy sintered at 1550 °C reaches 65.8 ± 1.5 HRA (Figure 8a) while the elastic modulus and compressive yield strength is 50.8 ± 3.9 GPa and 1058 ± 35.1 MPa (Figure 8b), respectively. This is superior to the mechanical properties fabricated by the cold-crucible levitation melting (compressive yield strength of 730 MPa [23]). The improvement in the compressive yield strength of the BMP sintered samples stems from two reasons. The first reason is that there is some oxygen introduced during the process of ball milling, which can also can be seen from the powders XRD results for the powders. In addition, compared with the samples fabricated by cold-crucible levitation melting, the PM-fabricated samples have smaller grains, uniform microstructure and less defects, which can also improve the strength.

### 3.5. In Vitro Biocompatibility

**Figure 9:**
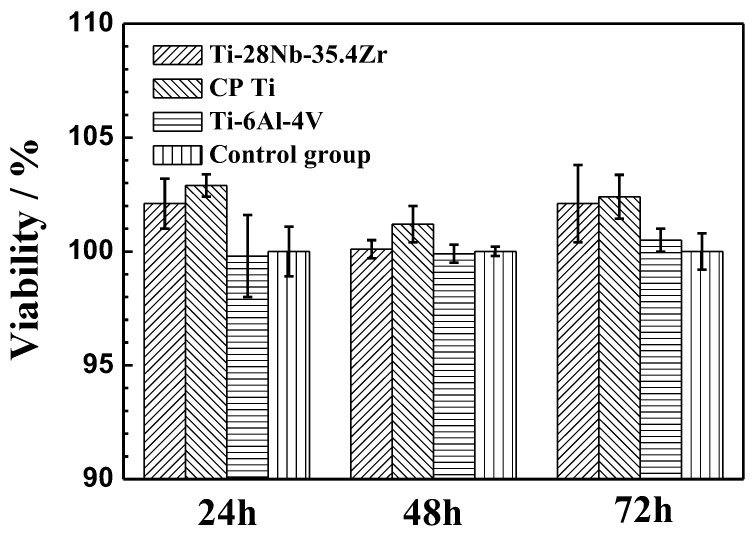
MTT result of MC3T3-E1 cells cultured with extracts of Ti-28Nb-35.4Zr, CP Ti and ELI Ti-6Al-4V.

In vitro biocompatibility of the Ti-28Nb-35.4Zr alloy sintered at 1550 °C by the milled powder was evaluated by cell tests. The CVR was calculated by Equation (2). The control group has a CVR of 100%, which is accepted as a reference for determining the cell viability radio of samples [34]. Figure 9 shows the results of CVR using MC3T3-E1 cells grown in Ti-28Nb-35.4Zr alloy, ELI Ti-6Al-4V and CP-Ti extracts for 1, 2 and 3 days. It can be seen that the average CVR value of all the experimental alloys was above 99%. The Ti-28Nb-35.4Zr alloy exhibits a slightly higher average CVR value than ELI Ti-6Al-4V while was similar to CP Ti. However there were no statistically significant differences among them (P > 0.05). According to ISO 10993-5 [31], the cytotoxicity grade of Ti-28Nb-35.4Zr alloy and CP Ti is 0–I grade while that of ELI Ti-6Al-4V is 0–I grade. Consequently, the PM-fabricated Ti-28Nb-35.4Zr alloy shows good in vitro biocompatibility to MC3T3-E1 cells.

**Figure 10:**
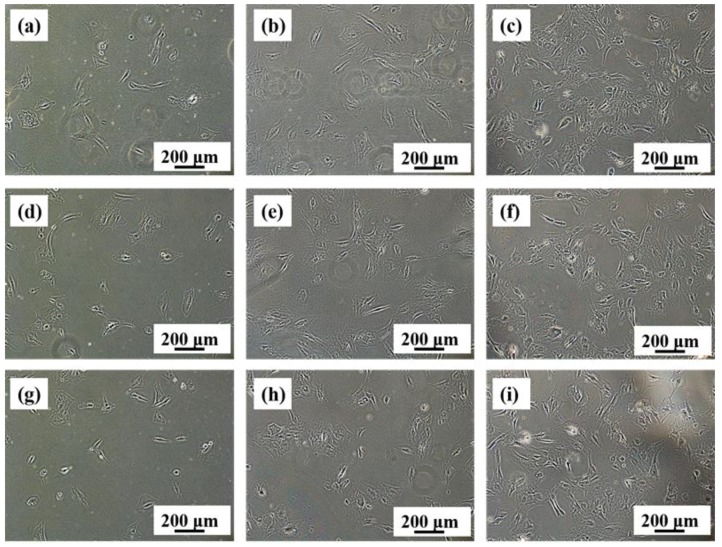
The number and morphology of MC3T3-E1 cells cultured with extracts of Ti-28Nb-35.4Zr ((**a**): 24 h; (**b**): 48 h; (**c**): 72 h), CP Ti ((**d**): 24 h; (**e**): 48 h; (**f**): 72 h) and ELI Ti-6Al-4V ((**g**): 24 h; (**h**): 48 h; (**i**): 72 h).

Figure 10 displays the number and morphology of MC3T3-E1 cells cultured with extracts of Ti-28Nb-35.4Zr ELI Ti-6Al-4V and CP-Ti for different periods (1 day, 2 days and 3 days). It can be seen that the morphologies of all cells are similar for different periods. After a 1-day culture, cells are basically adhered to the well and the shape is spindle. With a prolonged culture time, cells cultured by different Ti alloys become denser and stretched, although there are no significantly changes in MTT.

The in vitro biocompatibility of an implant lies not only in the toxicity of its alloys elements, but also in its corrosion resistance. Ti-Nb-Zr alloys are formed by the solid solutions of pure Ti, Nb and Zr, and maintain a high corrosion resistance and low toxicity [35]. Further, the corrosion resistance of Ti-Nb-Zr alloys is superior to that of CP Ti and ELI Ti-6Al-4V [36]. Spontaneously formed oxides, made up of TiO_2_, Nb_2_O_3_ and Zr_2_O_3_, will provide a bio-inert layer in aggressive body fluids [37]. The enrichment of TiO_2_, Nb_2_O_3_ and Zr_2_O_3_ on the surface suppressed the dissolution of Ti, Zr and Nb as ions. Hence, the Ti-28Nb-35.4Zr alloys show high cell viability during the MTT assays. Coupled with the low elastic modulus, high compressive yield strength and net-shape manufacturability, it thus suggests that PM-fabricated Ti-28Nb-35.4Zr alloy may hold promise for the development of next class of low-cost, highly-biocompatible, Ti-based biomedical alloys.

## 4. Conclusions

(1) Ti-28Nb-35.4Zr alloy with a high relative density and uniform microstructure can be obtained from milled powder via PM. After milling, the average particle sizes of powders was 15.2 μm, and when sintered at 1550 °C, the relative density reaches 98.1 ± 1.2%. The alloy fabricated by milled powders at 1550 °C is characterized by the single β phase.
(2) The alloys fabricated by the ball-milled powder at 1550 °C can achieve a high mechanical properties with the compressive yield strength of 1058 ± 35.1 MPa, the elastic modulus of 50.8 ± 3.9 GP, and the hardness of 65.8 ± 1.5 HRA. This is superior to the mechanical properties fabricated by the cold-crucible levitation melting technique.
(3) From in vitro cytotoxicity tests, the Ti-28Nb-35.4Zr alloy fabricated by milled powder at 1550 °C has no adverse effects on cell proliferation, and the cytotoxicity level is ranked as 0 grade, which is similar to the existing biomedical materials of CP Ti or ELI Ti-6Al-4V.
(4) Based on its demonstrated net-shape manufacturability by PM, high compressive yield strength, low elastic modulus and excellent in vitro biocompatibility, the PM-fabricated Ti-28Nb-35.4Zr can be considered as an attractive orthopedic implant alloy.
